# Inflammatory Pathogenesis of Post-stroke Depression

**DOI:** 10.14336/AD.2024.0203

**Published:** 2024-02-03

**Authors:** Xinyu Feng, Xiaojuan Ma, Juan Li, Qing Zhou, Yao Liu, Jingyi Song, Jiaqi Liu, Qingqing Situ, Lingyue Wang, Jingzhi Zhang, Facai Lin

**Affiliations:** School of Acupuncture and Tuina, School of Health and Rehabilitation, Nanjing University of Chinese Medicine, Nanjing, Jiangsu, 210023, China

**Keywords:** Post-stroke depression, Inflammatory mechanism, Stroke, Depression

## Abstract

Post-stroke depression (PSD) is a complex mood disorder that emerges in individuals following a stroke, characterized by the development of depressive symptoms. The pathogensis of PSD is diverse, with inflammation playing a vital role in its onset and progression. Emerging evidence suggests that microglial activation, astrocyte responses, nuclear factor κB(NF-κB) signaling, dysregulation of the hypothalamic pituitary adrenal (HPA) axis, alterations in brain-derived neurotrophic factor (BDNF) expression, neurotransmitter imbalances, adenosine triphosphate (ATP) and its receptors and oxidative stress are intricately linked to the pathogenesis of PSD. The involvement of inflammatory cytokines in these processes highlights the significance of the inflammatory pathway. Integrating these hypotheses, the inflammatory mechanism offers a novel perspective to expand therapeutic strategies for PSD.

During the occurrence of a cerebrovascular accident, a series of deleterious events occur in the brain, including inflammation and neurotoxicity, which can lead to stroke with or without reperfusion therapy [1]. Stroke is notorious for its high prevalence, mortality and morbidity rates, positioning it as the second leading cause of death and disability on a global scale [2]. It also ranks among the top four causes contributing to the disability-adjusted life years in the realm of neurological disorders [3]. Notably, about one-third of individuals who survive a stroke experience depression in the aftermath. PSD is a frequent aftermath of stroke, with its incidence ranging between 11% and 41% within the first two years post-event [4]. The clinical profile of PSD spans a spectrum from mood lows, social withdrawal, dwindling interests, emotional dullness and pessimism to sleep issues, fatigue, and in extreme cases, suicidal ideation [5]. PSD not only delays neurological recovery but also reduces the quality of life, exerting a significant impact on patients personally while also imposing a substantial burden on families and society. Extensive research indicates the involvement of inflammatory cytokines in the pathogenesis of PSD [6-9]. The pathogenesis of PSD is extremely complex, involving various hypotheses, including the immune cell hypothesis, HPA axis dysfunction hypothesis, neurotrophic factor hypothesis, neurotransmitter hypothesis, oxidative stress hypothesis, and more. Moreover, these hypotheses are not independent but intricately interconnected. After a stroke occurs, there is dysfunction in the HPA axis, accompanied by the emergence of oxidative stress [10]. Such disturbances trigger a cascade of events starting from neural cell activation, leading to inflammatory responses characterized by elevated levels of pro-inflammatory cytokines such as IL-1, IL-6, and tumor necrosis factor-alpha (TNF-α) [11]. Concurrently, it induces the production of neurotransmitters, ultimately resulting in hippocampal dysfunction and a decrease in the generation of 5-HT, NE, and DA, leading to the manifestation of PSD. (For specific connection pathways, please refer to Fig. 1.)

**Figure 1.**
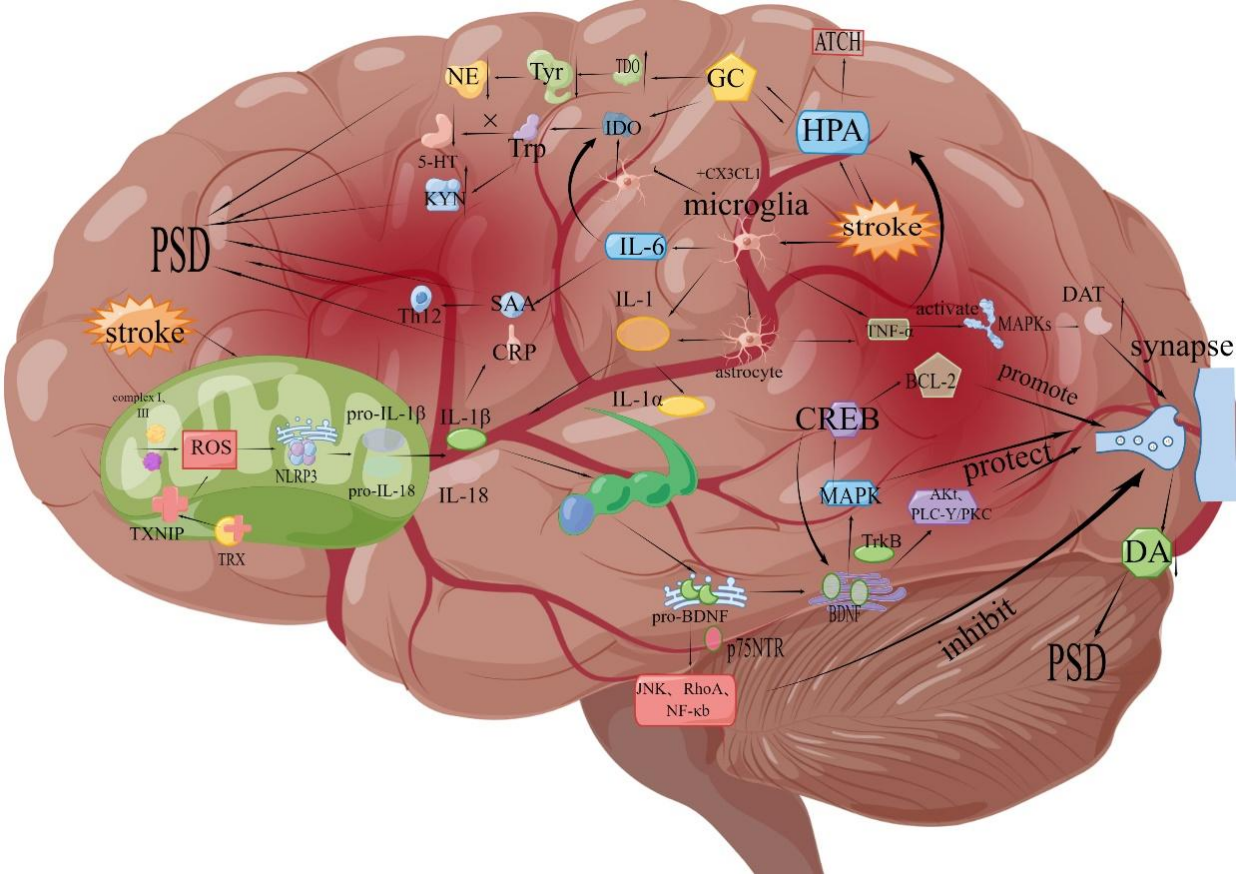
**Summary of the inflammation hypothesis in post-stroke depression**. After stroke onset, mitochondrial damage, HPA axis dysfunction, and microglial activation. The dysfunctional HPA axis promotes ATCH and GC production and affects TDO and IDO production, resulting in decreased NE and 5-HT production, increased KYN production and PSD. Activated microglia induce IL-1, IL-6, and TNF- α production, and can also activate the activation of astrocytes. Increased IL-6 participates in the generation of IDO thus leading to reduced 5-HT production, and activation of MAPKs by TNF- α from glial cells induces reduced DA production at synapses, leading to the appearance of PSD. The injured mitochondria produce large amounts of ROS, leading to the production of the proinflammatory factor IL-1 β and IL-18. Proinflammatory factors are involved in the induction of SAA and CRP and are closely related to PSD. IL-18 generated by mitochondrial damage impairs hippocampal function, leading to the production of BDNF precursors in the endoplasmic reticulum in the hippocampus, and hence the formation of mature BDNF in the Golgi apparatus. The resulting BDNF affects synaptic function through JNK, AKt, resulting in reduced DA production and PSD (by Figdraw).

## 1. Neuroinflammation in Stroke

Inflammation is a consequence of ischemic injury, serving a dual role in the aftermath of a stroke. Initially, it facilitates the clearance of necrotic tissue, setting the stage for the subsequent repair and remodeling of the affected brain regions. However, this inflammatory response can also negatively impact stroke prognosis by exacerbating tissue damage and propelling the progression of ischemic brain injury [12]. After a stroke, ischemic neurons release an array of mediators due to impaired blood flow, activation of leukocytes within the vasculature and the release of pro-inflammatory agents from the ischemic endothelium and brain parenchyma, including cytokines, chemokines, adhesion molecules and reactive oxygen species (ROS). These inflammatory mediators exacerbate tissue damage and hasten the evolution of ischemic brain injury. Intriguingly, under specific conditions, these inflammatory mediators can induce ischemic tolerance, offering a protective mechanism against cerebral ischemia [13]. The activation of the inflammatory response leads to the upregulation of cell adhesion molecules, cytokines and chemotactic factors, as well as the infiltration of white blood cells into ischemic tissue. In the early stages of cerebral ischemia, white blood cells are recruited by cell adhesion molecules expressed on endothelial cells, leading to overexpression of pro-inflammatory genes. In the later stages, these cells infiltrate into the parenchyma, significantly impacting the pathogenesis of ischemic brain injury [14]. This highlights the potential of anti-inflammatory treatments in mitigating stroke outcomes.

Dysfunction of the blood-brain barrier (BBB) is also a hallmark of stroke. Under normal physiological conditions, an intact BBB can effectively balance central and peripheral inflammatory mediators. However, when a stroke occurs, the BBB is compromised, allowing a large influx of peripheral inflammatory cells and mediators into the brain, exacerbating neuroinflammation [15]. Cerebral ischemia activates perivascular astrocytes. Due to the swelling of astrocytic end-feet and retraction of processes from blood vessels, gap junctions are opened, leading to the disruption of endothelial cells and resulting in BBB damage. The increase in endothelial vesicle number is considered a crucial indicator of BBB disruption [16]. In response to ischemic stimuli, activated astrocytes release various substances that exacerbate the BBB's permeability, further compromising its protective function [17]. Such BBB breakdown facilitates the influx of white blood cells into the brain, escalates cytokine production, and heightens the risk for cerebral edema and hemorrhagic transformation, complicating the post-stroke recovery process [18]. During the early stages of ischemic injury, the damage-associated molecular patterns (DAMPs) and cytokines expressed can enter the body circulation through the disrupted BBB. This induction can trigger immune responses in primary and secondary lymphoid organs, leading to systemic inflammatory response syndrome [13]. The breakdown of the BBB will cause a large number of immune cells to infiltrate into the brain, triggering an immune response. This immune activation is characterized by the release of signaling molecules from the injured tissue, invoking a potent immune reaction aimed at repairing the damage [19]. Furthermore, adhesion molecules on endothelial cells stimulate circulating white blood cells to adhere to activated endothelial cells, leading to microvascular blockade and the infiltration of immune cells into the brain parenchyma [20].

## 2. Neuroinflammation in PSD

Inflammation plays a crucial role in the reparative process following secondary brain injury and stroke. After the occurrence of acute stroke, both central and peripheral immune inflammatory responses are immediately activated. Subsequently, pro-inflammatory cytokines initiate and amplify the inflammatory response, leading to dysfunction of the adrenergic system, overactivity of the HPA axis, widespread activation of indoleamine 2,3-dioxygenase(IDO), accelerated depletion of serotonin(5-HT) in areas such as the left frontal and temporal cortices, ultimately resulting in the onset of depression [21-25]. The inflammatory underpinnings of PSD are closely linked to various cellular and molecular actors, including microglial cells, astrocytes, the NF-κB, the HPA axis, BDNF, neurotransmitters, ATP, C-Reactive Protein (CRP) and oxidative stress. These components interplay intricately within the inflammatory landscape, highlighting the complexity of PSD's pathogenesis. A comprehensive understanding of these mechanisms is essential for devising effective strategies for both the prevention and management of PSD, underscoring the need for targeted therapeutic interventions that address the multifaceted nature of post-stroke inflammation and its contribution to depressive symptomatology.

### 2.1 Inflammatory Mediators in PSD

#### 2.1.1 Role of Cytokines in PSD

Cytokines, critical signaling proteins, facilitate intercellular communication and are chiefly produced by immune cells such as monocytes, macrophages and lymphocytes. Their role extends beyond the immune and hematopoietic realms, influencing the nervous and endocrine systems profoundly. In the setting of PSD, a notable elevation in pro-inflammatory cytokines like IL-1, IL-6 and TNF-α has been documented. These cytokines have the capacity to directly affect brain regions pivotal for emotional regulation, potentially leading to the manifestation of depressive symptoms.

IL-1 is a common factor in the cytokine family, with two subtypes, IL-1α and IL-1β. IL-1β, in the context of ischemic stroke, is more prominently involved in driving inflammatory responses and contributing to neurodegenerative alterations. IL-1β is also a key factor in inducing depressive-like behavior in response to acute and chronic stress [26]. Furthermore, IL-1β plays a crucial role in modulating synaptic plasticity and associated behavioral outcomes, significantly impacting cognitive functions related to the hippocampal region [27]. The NOD-like receptor thermal protein domain associated protein 3(NLRP3) inflammasome's involvement in the regulation of IL-1β secretion by microglial cells underscores its contribution to the inflammatory processes linked to depression. Clinical investigations have highlighted elevated serum IL-1β levels in individuals with PSD compared to their non-PSD counterparts [28]. Additionally, the presence of the -511 T allele has been associated with higher IL-1β levels shortly after a stroke, indicating a potential link to PSD, although this association appears to diminish over time [28]. This intricate interplay between cytokines and their impact on neural pathways underscores the complex pathophysiological landscape of PSD, where inflammatory mediators play a pivotal role in shaping the neuropsychiatric outcomes following a stroke.

Within the central nervous system (CNS), IL-6 is synthesized by an array of cells including neurons, astrocytes, microglial cells, and endothelial cells, playing a vital role in the inflammatory response linked to PSD Elevated serum IL-6 levels in the acute phase of ischemic stroke are independently associated with the occurrence of depression at two weeks and one-year post-stroke[29]. This correlation is further emphasized by findings that higher serum IL-6 levels are directly related to the intensification of PSD's physical manifestations [30]. Moreover, IL-6, alongside IFN-γ, has been shown to regulate the expression of IDO, an enzyme that hinders the conversion of Trp into 5-HT. This regulatory effect on the tryptophan-serotonin pathway elucidates a biochemical link between inflammation and the neuropathology of depression following a stroke, thereby contributing to the onset and progression of PSD [31]. This intricate interplay between IL-6 and neurochemical processes in the brain underscores the multifaceted nature of PSD's pathogenesis, highlighting the importance of understanding cytokine activity in devising effective treatment strategies for this condition.

The tumor necrosis factor (TNF) family, encompassing TNF-α and TNF-β, plays a pivotal role in the realms of oncology and immunology, drawing significant research attention. Under internal and external stimuli, both astrocytes and microglial cells can secrete TNF-α, participating in cellular signal transduction, inflammation, and processes such as apoptosis. As a pro-inflammatory factor, TNF-α has been implicated in the pathogenesis of depression. Studies have demonstrated elevated levels of TNF-α in the serum of PSD patients, with a positive correlation to depression severity, highlighting its potential role in PSD's pathophysiology [32]. TNF-α's overexpression, mediated by its receptors on neuronal membranes, affects synaptic signaling and neuroplasticity, thereby influencing neuronal functionality and contributing to PSD, influencing the development of PSD [33]. Therefore, the detection and targeted intervention of concentrations of IL-1β, IL-6 and TNF-α post-stroke may contribute to the diagnosis and treatment of PSD.

**Figure 2.**
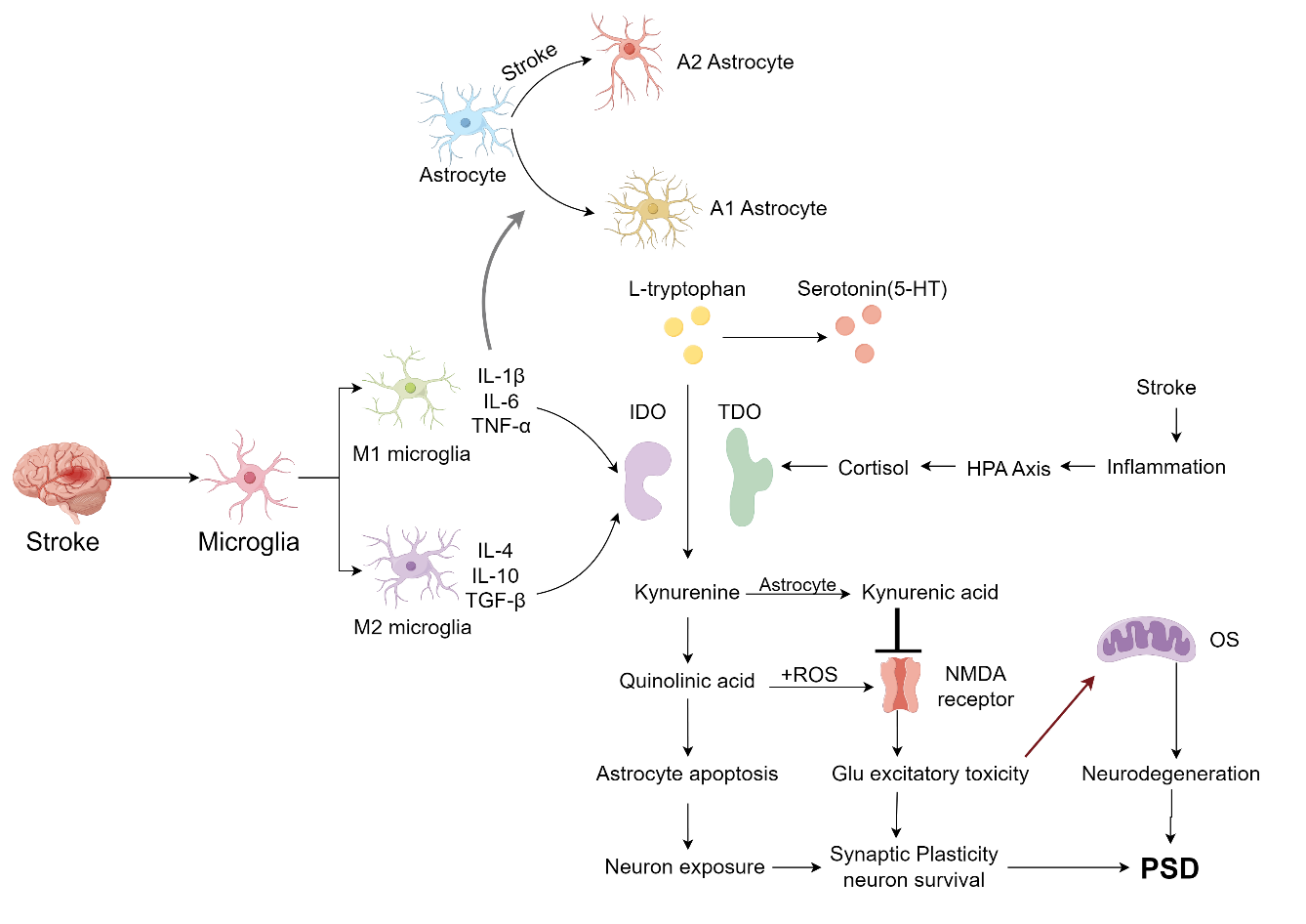
**Inflammatory Mediators in PSD**. During a stroke event, microglia differentiate into M1 and M2 phenotypes, while astrocytes differentiate into the A2 type. M1 microglia release pro-inflammatory cytokines, which induce the differentiation of astrocytes into the A1 type. M2 microglia, on the other hand, release anti-inflammatory cytokines. The expression of pro-inflammatory cytokines can lead to an increase in IDO synthesis. Stroke-induced inflammatory cytokines act on the HPA axis, and elevated cortisol levels increase TDO activity. Under normal conditions, L-tryptophan is converted to 5-HT. However, in the presence of IDO and TDO, L-tryptophan can be converted into kynurenine and QA, inhibiting its conversion to 5-HT. Furthermore, QA can induce apoptosis in astrocytes, leading to neuronal exposure and affecting neuronal survival. The combination of QA and reactive oxygen species can affect NMDA receptor function. Kynurenic acid, which can be converted from kynurenine in astrocytes, inhibits NMDA receptor function, thereby increasing glutamate excitotoxicity. This leads to a reduction in synaptic plasticity and neuronal survival, resulting in PSD. Glutamate excitotoxicity, involved in oxidative stress responses, can lead to neurodegenerative changes and also contribute to the development of PSD (by Figdraw).

Conversely, IL-10, an anti-inflammatory cytokine, is associated with stroke outcomes and quality of life, acting as a protective factor against PSD [8]. Lower levels of IL-10 post-stroke have been linked to an increased risk of PSD, emphasizing the protective role of anti-inflammatory cytokines in this context [34]. However, some studies have not found a direct correlation between IL-10 levels and PSD, suggesting variability in cytokine responses among individuals [35]. The imbalance between pro-inflammatory and anti-inflammatory cytokines, such as IL-1β, IL-6, TNF-α and IL-10, may play a crucial role in PSD's chronic development, pointing towards the importance of maintaining cytokine homeostasis in preventing and treating PSD [7] (Refer to Fig. 2 for a visual representation of cytokine interactions in PSD).

#### 2.1.2 Chemokines and Their Impact on PSD

Chemokines, triggered by inflammatory cytokines, play a crucial role in mediating immune responses within the CNS, particularly in the context of PSD. These molecules function as chemoattractant, guiding monocytes, neutrophils and other immune cells from the bloodstream to sites of inflammation or injury. Within the immune system, chemokines orchestrate the migration of these cells to areas requiring an immune response.

Specifically, CC-class chemokine ligands (CCL), including CCL2, CCL7, CCL8, CCL12 and CCL13, are instrumental in directing pro-inflammatory cells towards inflamed or damaged brain regions, playing a significant role in the neuroinflammatory process associated with PSD [36, 37]. For instance, CCL2 acts as chemical inducers, can promote the occurrence of inflammatory events in the CNS [38]. The interaction between CCL2 and its receptor, CCR2, has been implicated in promoting neuroinflammation, reducing 5-HT release and eliciting depressive-like behaviors in animal models, shedding light on the potential mechanisms underlying PSD [39]. Moreover, the CX3C class chemokine ligand 1 (CX3CL1) present on neurons interacts with the CX3C chemokine receptor 1 (CX3CR1) on microglial cells, maintaining these immune cells in a quiescent state and preventing their undue activation [40]. This interaction highlights the delicate balance between neuroinflammatory and neuroprotective forces within the brain, and its disruption could contribute to the pathogenesis of PSD. Understanding the role of chemokines in neuroinflammation provides valuable insights into the intricate interplay between the immune system and neurological health, particularly in the aftermath of a stroke, offering potential therapeutic targets for mitigating PSD.

#### 2.1.3 Prostaglandins and Leukotrienes in PSD

Following a stroke, the activation of microglial cells and astrocytes leads to the production and release of various pro-inflammatory mediators, including a significant number of prostaglandins. These prostaglandins, including prostaglandin D2 (PGD2), prostaglandin E2 (PGE2) and cyclic prostaglandins such as prostaglandin J2 (PGJ2) and prostaglandin A2 (PGA2), play a crucial role in the response to cerebral ischemia and the development of PSD [41, 42]. Notably, Prostaglandin E1 (PGE1) is recognized for its neuroprotective properties against cerebral ischemia [43], whereas PGE2 exhibits a multifaceted role by modulating excitotoxic damage, promoting neuroinflammation, exacerbating brain infarction, and contributing to neurological impairments [44].

Leukotrienes, another class of pro-inflammatory molecules primarily produced by macrophages and neutrophils, are also crucial in the context of PSD. They have been linked to an elevated risk of stroke and play a significant role in mediating brain injury and the ensuing inflammatory responses post-stroke [45, 46]. The increase in leukotriene B4 (LTB4) levels, in particular, is associated with neutrophil infiltration into the brain, exacerbating injury and inflammation. Research suggests that inhibiting leukotriene production can mitigate brain damage and improve outcomes following cerebral ischemia, with the inhibition of 5-lipoxygenase (5-LOX) being notably effective. This inhibition leads to an increase in anti-inflammatory cytokine IL-10 levels and a reduction in pro-inflammatory cytokines within the CNS, thereby aiding in the alleviation of PSD symptoms [47, 48]. Understanding the roles of prostaglandins and leukotrienes in the aftermath of a stroke provides valuable insights into the complex interplay between inflammatory processes and the development of PSD. Targeting these pathways may offer potential therapeutic strategies for mitigating the neuropsychiatric complications associated with stroke recovery.

### 2.2 Glial activation and neuroinflammation

#### 2.2.1 Inflammation and Microglial Cells

In the event of trauma or inflammation within the CNS, microglial cells—acting as the brain's resident immune sentinels—detect abnormal signals through their surface membrane receptors and ion channels [49]. This detection triggers a cascade of molecular responses, leading to the activation of microglial cells. Once activated, these cells display increased phagocytic activity and undergo significant morphological changes, resembling an amoeboid shape, to effectively respond to the injury [50]. Microglial cells play a pivotal role in resolving neuroinflammation and fostering neural recovery by engulfing and clearing cellular debris. In the early stages of injury, microglial cells release anti-inflammatory factors to repair damaged neurons However, if the injury persists, microglial cells shift towards a pro-inflammatory state, secreting factors that not only amplify inflammation but also exacerbate neuronal damage. These cells can adopt two distinct phenotypes: the M1 phenotype, associated with neurotoxic effects, and the M2 phenotype, known for its neuroprotective actions [51, 52]. It's worth noting that polarized activation, M1 or M2 phenotype, is a simple and ideal approach for providing in vivo therapeutic strategies and inhibiting microglial cells. This can be used as a strategy for treating neuroinflammatory diseases [53]. The cascade of pro-inflammatory mediators and reactive species released by activated M1 microglia can disrupt astrocyte functionality, diminish neurotrophic support, and impede hippocampal neurogenesis, crucial for brain repair and cognitive functions [54]. This disruption precipitates damage in critical mood-regulating areas such as the frontal cortex, hippocampus, and amygdala, potentially triggering PSD. Emerging perspectives highlight the integral role of the immune response—encompassing astrocytes, inflammatory cells, and microglia—in the onset of PSD following a stroke, underscoring the intricate interplay between the brain's immune and neural systems [55].

In the aftermath of PSD, microglial cells are known to swiftly converge on the lesion site, intensifying tissue damage through the release of inflammatory cytokines and neurotoxic substances. Conversely, they also contribute to tissue repair and remodeling by eliminating debris and secreting anti-inflammatory cytokines and growth factors, underscoring their dual role in the brain's response to injury [56]. Additionally, the involvement of purinergic receptor signaling in microglia underscores the complexity of neuroinflammatory pathways in PSD, suggesting potential therapeutic targets [57]. Recent findings link PSD to a reduction in miR34b-3p levels in hippocampal neurons, leading to enhanced microglial activation and depressive-like behaviors in animal models. Targeting the miR34b-3p and eIF4E axis may offer novel therapeutic strategies for mitigating PSD symptoms [58].

Microglia, the brain's immune cells, exhibit a dual nature by secreting both healing anti-inflammatory and potentially harmful pro-inflammatory cytokines and chemokines. When activated, these cells trigger the expression of inflammatory genes through complex signaling pathways, including Janus kinase/signal transducer and activator of transcription 3 (JAK/STAT3), glycogen synthase kinase 3β (GSK-3β), Phosphatase and tensin homology deleted on chromosome ten (PTEN) and protein kinase B (Akt). This understanding opens the door to targeting microglial activation as a therapeutic strategy for PSD, aiming to modulate these critical signaling cascades [59]. The concept of microglial polarization, distinguishing between the pro-inflammatory M1 phenotype and the anti-inflammatory M2 phenotype, provides a framework for developing in vivo treatments for neuroinflammatory conditions [60]. Among pharmacological interventions, fluoxetine, a well-known selective serotonin reuptake inhibitor (SSRI), has been shown to inhibit the activation of NLRP3 inflammasomes in microglial cells, offering relief from depressive-like symptoms [61]. Minocycline, another compound, exerts its antidepressant effects predominantly by dampening microglial activation and lowering inflammatory mediator levels [62].

Recent studies have linked chronic stress-induced depressive behaviors in ischemic stroke models to the activation of microglia in the hippocampus, an area crucial for mood and memory [63]. Strategies that inhibit microglial activation, boost hippocampal levels of BDNF and increase dendritic spine density have shown promise in mitigating PSD-like behaviors in animal models [64]. RNA sequencing revealed a significant decrease in miR34b-3p in the hippocampus of PSD mice. miR34b-3p is expressed in neurons and can inhibit the translation of eIF4E. Silencing eIF4E can deactivate microglial cells, suppress neuroinflammation, and alleviate depressive-like behavior in PSD mice. Therefore, promoting the expression of miR34b-3p and silencing eIF4E emerge as potential therapeutic targets for treating PSD [65].

#### 2.2.2 Inflammation and Astrocytes

Astrocyte activation is a pivotal response to ischemic stroke, with their activation playing a significant role in the neuroinflammatory landscape [66]. Following a stroke, activated microglia secrete a combination of IL-1α, TNF-α and C1q, prompting astrocytes to adopt a neurotoxic phenotype, which contributes to the complexity of the neuroinflammatory response [64]. Astrocytes, with their unique structural and functional attributes, are integral to neuroprotection. They regulate synaptic activities, aid in synaptic maturation and elimination, and manage neurotransmitter dynamics within synaptic clefts, thus influencing neuronal excitability [67]. After a stroke, astrocytes are activated and effectively demarcate the injury site, limiting leukocyte infiltration, promoting hemoglobin repair, and supporting neuronal survival [68]. Astrocytes primarily influence synaptic transmission through the release of neurotransmitter, modulating synaptic excitability, plasticity and network synchronization. This modulation plays a a crucial role in the network dysregulation observed in neuropsychiatric conditions. Specifically, the release of glutamate by astrocytes is pivotal in regulating synaptic transmission and is implicated in the excitotoxicity observed in various neurological disorders [69]. Inflammatory mediators can disrupt the extracellular glutamate balance by impairing the glutamate clearance capacity of both microglia and astrocytes, leading to an accumulation of glutamate in the synaptic cleft and reduced intracellular glutamate in neurons. This imbalance can excessively activate NMDA receptors, inducing excitotoxicity and contributing to neuronal degeneration, which may precipitate PSD development [70]. Astrocytes also play a crucial role in the glutamate-glutamine cycle, facilitating neurotransmitter synthesis and supporting synaptic function. Disruptions to this cycle after stoke can lead to an imbalance in glutamate homeostasis, further exacerbating excitotoxicity [71]. Moreover, the reduction in the expression of the glutamate transporter GLT-1 in astrocytes within the PSD context can hinder glutamate metabolism and synaptic formation, adding another layer to the complex pathology of PSD [72]. Additionally, astrocytic glutamate transporters are essential for clearing excess glutamate, preventing excitotoxicity and maintaining neuronal integrity. Notably, the expression levels of astrocytes in PSD models are significantly lower than in control groups, highlighting the potential impact of astrocyte dysfunction on PSD pathology [73].

Certain astrocytic derivatives exhibit neuroprotective capabilities that can mitigate brain damage and potentially prevent the onset of PSD, notably including mesencephalic astrocyte-derived neurotrophic factor (MANF) and insulin-like growth factor-1 (IGF-1). MANF, produced by midbrain astrocytes, offers neuroprotection, promotes neuroregeneration and supports the survival of dopaminergic neurons, highlighting its multifaceted therapeutic potential [74]. In ischemic conditions, both neurons and astrocytes exhibit elevated MANF expression, which can reverse behavioral deficits induced by stroke, suggesting its critical role in neuroprotection. The absence of MANF is linked to increased infarct volumes in ischemic brain models, underscoring its importance in neural integrity [75]. The administration of recombinant forms of cerebral dopamine neurotrophic factor (CDNF) or MANF post-stroke has been shown to enhance recovery of neurological function, presenting a promising therapeutic strategy for addressing stroke-induced limb dysfunctions [76]. IGF-1 plays a significant role in reducing cytotoxic death, fostering the growth and maturation of new neurons and glial cells within injured tissues, stimulating neurovascular regeneration during the chronic stages of cerebral infarction, thus contributing to brain repair mechanisms [77]. After a stroke, the brain tissue experiences hypoxia and glucose deprivation, triggering astrocyte activation. Aromatase activity in these reactive astrocytes leads to the synthesis of 17β-estradiol (E2), a steroid hormone with noted neuroprotective properties. Neuron-derived E2 is crucial for stimulating activated astrocytes to produce IGF-1, further amplifying its neuroprotective effects [78, 79]. Recent studies have demonstrated that administering IGF-1 three days post-stroke significantly improves tissue recovery and sensory-motor functions, indicating its therapeutic efficacy in stroke rehabilitation [80].

The intricacies of astrocytic signaling pathways, particularly those activated by peripheral inflammation and the role of calcium (Ca^2+^) signaling in essential cellular functions within astrocytes, remain somewhat elusive in the context of depression's pathogenesis. Notably, Ca^2+^ signaling in astrocyte-mediated processes has not been directly explored in relation to depression, marking a significant gap in our understanding [81].

Repetitive transcranial magnetic stimulation (rTMS) treatment shows promise in ameliorating depression-like behaviors in rat models. This therapeutic effect is attributed to the activation of the phosphoinositide 3-kinase (PI3K)/Akt/cAMP response and cAMP-response element binding protein (CREB) signaling pathway. By upregulating the expression of the glutamate transporter GLT-1, rTMS reverses its reduction on astrocytes, thereby diminishing glutamate accumulation in the synaptic cleft. This contributes to the mitigation of synaptic plasticity damage and neuronal apoptosis, preserving the equilibrium between glutamatergic and gamma-aminobutyric acid (GABA) ergic systems and significantly curtails hippocampal neuronal activity impairment [81].

Resveratrol, a polyphenolic compound, enhances astrocytic AMP-activated protein kinase (AMPK) activity, suppresses GSK-3β function, mobilizes energy, supplies ATP to neurons, reduces reactive ROS levels and increases oligodendrocyte survival. These actions collectively aid in maintaining brain homeostasis post-stroke, positioning resveratrol as a viable candidate for addressing PSD symptoms and a potential future therapeutic agent [81]. Valproic acid, a drug commonly utilized in epilepsy management, has been shown to attenuate astrocyte activation and the production of extracellular matrix proteins. Its neuroprotective properties during the ischemic stroke recovery phase are believed to be linked to the enhanced expression of acetylated histone 3, 4 and heat shock protein 70.1B in astrocytes, highlighting its potential utility in neurorehabilitation [82].

Betaine, known for its neuroprotective effects, not only inhibits the activation of microglia and astrocytes but also facilitates the transformation of microglia from pro-inflammatory M1 state to anti-inflammatory M2 state and astrocytes from a harmful A1state to a beneficial A2 statel. This dual modulation underscores betaine's therapeutic potential in neuroinflammatory and neurodegenerative conditions, including PSD [83].

#### 2.2.3 Endothelial Cells and Blood-Brain Barrier Integrity

In the aftermath of a stroke, the microvasculature within affected brain regions prominently displays an inflammatory profile, predominantly marked by endothelial cell activation, compromised BBB functionality and the engagement and permeation of leukocytes [12]. Elevated miR-22 levels in individuals with PSD may inflict damage upon endothelial cells, compromise the integrity the BBB disrupt the cohesive interaction between endothelial cells and astrocytes and precipitate microvascular brain injuries. Further investigations reveal that structural impairments within the brainstem and deeper neural circuits are tied to anomalies in serotoninergic transmission, playing a significant role in pathological landscape of PSD [84].

Endothelial cells constitute the primary barrier encountered by leukocytes on their way to tissues. The process of leukocyte extravasation into inflammatory sites necessitates the activation of a spectrum of adhesion molecules and signaling cascades in both leukocytes and endothelial cells [85]. The intricate process of leukocyte recruitment, crucial in inflammation and immune responses, encompasses stages such as rolling, slow rolling, adhesion, spreading, intravascular crawling, transendothelial migration and eventual migration to the site of inflammation. Within this recruitment cascade, β2 integrin, a leukocyte-specific integrin, plays a key role in decelerating cell rolling and facilitating most steps of the leukocyte recruitment process [86]. After a stroke, the invasion of white blood cells from the periphery can exacerbate cerebral no-reflow phenomena or further neuronal damage through various mechanisms. This includes the elicitation of vasoactive substances like leukotrienes and prostaglandins, which induce vasoconstriction and extend platelet aggregation, culminating in heightened neuronal injury [87]. Consequently, mitigating the recruitment of white blood cells holds the potential to reduce brain tissue damage, offering valuable insights for the clinical management of neural injuries following a stroke.

The BBB is essential for maintaining CNS homeostasis by regulating the entry of substances from the bloodstream into the brain parenchyma through the expression of tight junction protein complexes and various transport proteins [88]. Disruption in the BBB's integrity, especially in depression, may be precipitated by the absence of characteristic endothelial transport mechanisms, leading to early BBB breakdown and exacerbating depressive pathologies through multiple pathways. Stroke-induced brain vascular damage, characterized by the dismantling of tight junction proteins, dysregulation of transport proteins, disruption of intracellular transport and inflammatory damage, underscores the primary pathological shifts observed in such cerebral insults [89]. In post-stroke conditions, the degradation of proteins critical for maintaining tight junction integrity is exacerbated by inflammatory cytokines that activate Rab7a. This prompts the selective degradation of these junctional proteins, resulting in BBB dysfunction, and magnifying cerebral damage [90]. This suggests that PSD may originate from stroke-induced neuronal damage, which subsequently triggers mechanisms to protect the BBB against further immune cell infiltration and microglial activation [91]. Clinically, the enhanced expression of tight junction proteins is associated with reduced BBB permeability and a neuroprotective effect, whereas diminished levels of these proteins compromise the barrier's integrity, leading to neurological symptoms [92].

The therapeutic challenge in PSD lies in the reduced permeability of BBB, which hinders the efficacy of drugs intended for neural nutrition by blocking their brain access. Thus, treatment strategies for PSD could include modulating the inflammatory response, selectively opening the BBB and administering drugs that support neural nutrition [91].

Experimental approaches, such as early intraventricular injection of gene therapy vectors like rAAV-VEGF and rAAV-Ang1 aim to mitigate BBB dysfunction by controlling VEGF-mediated vascular leakage, Left unchecked, increased BBB permeability could elevate the risk of hemorrhagic transformation post-stroke [93]. Moreover, the association of the depressive state with increased inflammatory molecules and oxidative stress-induced vascular damage highlights the potential benefits of physical exercise. Exercise has been documented to boost antioxidant capacity, mitigate oxidative stress and stimulate anti-inflammatory cytokine production, thereby potentially regulating BBB permeability in PSD through diverse molecular and cellular pathways [82, 94].

### 2.3 Acute phase proteins

The acute phase reaction (APR) is a rapid systemic response to stressors such as inflammation and tissue damage, marked by significant changes in serum components, notably the levels of CRP and Serum Amyloid A (SAA). SAA, similar to CRP, is an acute-phase plasma protein predominantly produced by the liver and stimulated by inflammatory cytokines like IL-1β and IL-6 [95].

#### 2.3.1 Serum amyloid A

SAA serves as a key acute-phase protein involved in the chemotaxis and recruitment of inflammatory cells, with the SAA family being particularly prominent during APR [96].

The SAA family, which plays a crucial role in the acute-phase response, includes the inducible Acute-Phase SAA (ASAA) encoded by genes SAA1 and SAA2, non-functional pseudogenes SAA3, and the constitutively expressed C-SAA encoded by SAA4. The promoter regions of SAA1 and SAA2 genes are packed with recognition sequences for key transcription factors such as NF-κB, IL-6, along with sites for RAS activation factors. This setup makes them highly responsive to pro-inflammatory cytokines like IL-1β, IL-6 and TNF-α. This responsiveness boosts inflammatory responses, potentially delaying recovery from diseases [97]. Recent research highlights elevated SAA1 levels in the plasma of patients with clinical depression compared to controls, indicating a potential link between SAA levels and depressive states [98]. Furthermore, SAA's role in augmenting Th17 cell production may also contribute to depressive-like behaviors in animal models [99].

Triggered by cytokines such as IL-6, IL-1β and TNF-α, ASAA can modulate the microglial phenotype, encouraging the expression of pro-inflammatory M1 characteristics while suppressing the anti-inflammatory M2 traits [100]. This modulation is pivotal in the pathology of PSD, as microglia stimulated by SAA amplify disease progression by secreting IL-1β, IL-18 and inducing pyroptosis [101]. The presence of SAA in both central and peripheral nervous systems can activate microglia, fostering a pro-inflammatory environment conducive to depressive symptomatology [102]. Following injury, SAA's mRNA and protein are observed alongside astrocytes and microglia across in various brain regions, further implicating it in the neuropathology of PSD [103].

Intriguingly, pharmacological interventions targeting known SAA receptors like FPRL1, RAGE, and TLR2/4 on human carotid artery endothelial cells have shown potential in mitigating SAA-induced pro-inflammatory gene expression, providing a promising avenue for PSD therapeutic strategies [104]. This approach underscores the significance of understanding SAA's role in PSD and its potential as a target for innovative treatments.

#### 2.3.2 Dynamics of C-Reactive Protein in PSD

C-Reactive Protein (CRP), a pivotal biomarker for inflammation, is intricately linked with the regulatory and amplification processes of inflammation [105, 106]. Its association with PSD has sparked significant interest, identifying CRP as a prognostic indicator for acute ischemic stroke outcomes [107, 108]. Observations indicate a sustained elevation of CRP levels in stroke survivors up to 18 months post-event, although this elevation does not correlate directly with depression diagnoses [109]. However, emerging studies [110, 111] suggest a tangible link between CRP levels and depression, reinforced by meta-analyses revealing heightened CRP levels during stroke's acute phase as a predictor for increased PSD risk [112].

The linkage between CRP and stroke outcomes extends into the genetic realm, with variations within the CRP gene implicated in stroke vulnerability and prognoses [113, 114]. Detailed genetic analyses have identified a significant correlation of rs3093059 and rs3091244 polymorphisms with ischemic stroke, underscoring the CRP gene's role in stroke susceptibility. Such genetic alterations could potentially modulate the risk associated with ischemic strokes [115]. Furthermore, specific single-nucleotide polymorphisms (SNPs) within the CRP gene influence serum CRP levels, with certain hereditary SNPs like the A allele of rs1417938 and the C allele of rs1205 being more common among individuals with a familial predisposition to depression [116, 117]. Emerging research, including work by Otsuka et al., highlights how genetic variations in CRP can elevate pro-inflammatory molecules, leading to neuroinflammation, a critical factor in the pathogenesis of PSD [118, 119]. The association of CRP's rs2794520 and rs1205 C/T genotype with an elevated risk of early-onset PSD further emphasizes this point. Additionally, the G allele of rs3091244 has been identified as a risk factor for early-onset PSD, whereas the A allele serves as a protective element [22]. Despite these findings, the exploration of SNP sites related to inflammatory molecules is not exhaustive. Future large-scale genome-wide association studies are poised to uncover more genetic risk SNPs linked to early-onset PSD, providing a broader understanding of the genetic underpinnings of this condition [22].

The consensus within the scientific community emphasizes a strong connection between CRP levels and PSD, leading to three prevailing hypotheses. First, elevated post-stroke serum CRP levels might directly precipitate depression. Second, the surge in serum CRP levels post-stroke could indirectly trigger depression through yet-to-be-unveiled mechanisms. Third, polymorphisms within the CRP gene are intricately linked with the emergence of PSD. These perspectives offer a comprehensive view of CRP's multifaceted role in PSD, paving the way for nuanced approaches to understanding and addressing this complex condition.

**Figure 3.**
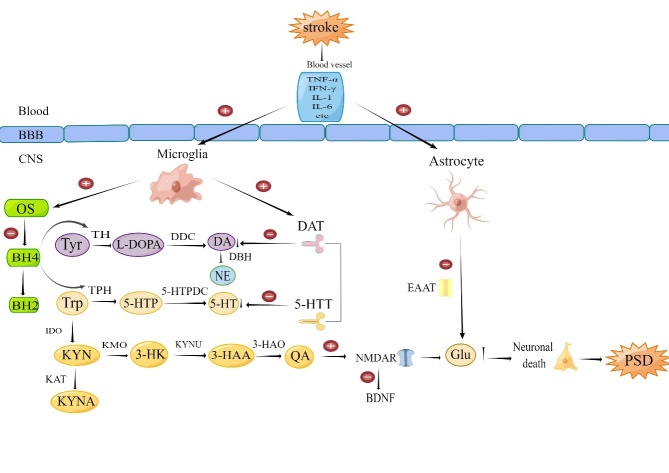
**Effects of inflammatory mediators on neurotransmitter systems**. Pro-inflammatory cytokines cause PSD by interfering with the synthesis of neurotransmitters such as 5-HT, DA, NE. They reduce the production of neurotransmitters by directly affecting the metabolic pathways of tyrosine and tryptophan. BH4 is a coenzyme factor of TH and TPH, and pro-inflammatory cytokines can also reduce coenzyme factor BH4 to BH2 by activating oxidative stress, thus further affecting neurotransmitter synthesis. In addition, they induce neurotoxicity by increasing the production of QA, which can inhibit and reduce the expression of BDNF, and also activate NMDAR, which causes glutamate to be released in excess, leading to nerve cell death, and ultimately promote PSD development. Proinflammatory cytokines can also directly release activate astrocytes to glutamate in excess and participate in the formation of PSD (by Figdraw). Abbreviations: Tyr, Tyrosine;TH, Tyrosine hydroxylase; L-DOPA, 3, 4-dihydroxyphenylalanine; DDC, Dopamine decarboxylase; DBH, Dopamine β-hydroxylase; DA, dopamine; DAT, Dopamine transporter; TPH, tryptophan hydroxylase; 5-HTP, 5-hydroxytryptophan; 5-HTPDC,5-hydroxytryptophan decarboxylase; 5-HT,5-hydroxytryptamine or serotonin; 5-HTT,5-hydroxytryptamine transporter; KYN, kynurenine; KMO, kynurenine 3-monooxygenase; 3-HK, 3-hydroxykynurenine; KYNU, kynureninase; 3-HAA, 3-hydroxyanthrenillc acid; 3-HAO: 3-hydroxyanthranilate 3,4-dioxygenase; QA, quinolinic acid; KAT, kynurenine aminotransferase I-III; KYNA, kynurenic acid;EAAT,Excitatory Amino Acid Transporter.

### 2.4 Effects of inflammatory mediators on neurotransmitter system

Inflammatory mediators significantly affect neurotransmitter systems, playing a pivotal role in the pathogenesis of PSD, particularly concerning monoamine and amino acid neurotransmitters. A deficiency in monoamine neurotransmitters, including 5-HT, dopamine (DA) and norepinephrine (NE) is a notable factor in PSD development. Serotonin, a key regulator of mood, sleep and memory, is synthesized from Trp. Pro-inflammatory cytokines can accelerate Trp metabolism through the activation of IDO, reducing 5-HT synthesis and increasing neurotoxic quinolinic acid (QA) production, thus contributing to PSD [120]. Interestingly, the absence of IDO gene abnormalities prevents depression the onset of even under inflammatory stimuli [121](Shown in Fig. 3). Studies examining urinary urea/tryptophan levels in stroke survivors have not found a direct correlation with depressive symptoms, suggesting complex underlying mechanisms [123]. However, interventions such as Cang-Ai Volatile Oil (CAVO) have shown potential antidepressant effects by modulating the IDO-mediated Trp degradation pathway and inhibiting inflammatory responses, thereby restoring the serotonin system [124]. Selective Serotonin Reuptake Inhibitors (SSRIs), the primary antidepressants, have been shown to lower future depression risk post-stroke [125]. Additionally, combination of inhibitors of 5-HT transport and kynurenine-3-monooxygenase counteract inflammation-induced delays in monoamine neurotransmission, improving depressive symptoms [126]. DA, essential for emotion and mood regulation, is synthesized from tyrosine (Tyr) with tetrahydrobiopterin (BH4) as a critical cofactor. Cytokine-induced oxidative stress (OS) can convert BH4 to BH2, diminishing DA synthesis [127]. Inflammatory states can also decrease the expression of Vesicular Monoamine Transporter 2 (VMAT2), crucial for neurotransmitter storage and release, resulting in reduced DA availability [128]. Moreover, inflammation affects DA Transporter (DAT) expression and function, with pro-inflammatory cytokines enhancing DAT reuptake and expression through mitogen-activated protein kinases (MAPKs) activation, thereby decreasing synaptic DA [128]. In an animal study, depressive-like behaviors were noted in mice following the induction of neuroinflammation via lipopolysaccharide (LPS) injections into the bilateral nucleus accumbens (NAc). This observation suggests that D3R expression within the NAc plays a critical role in moderating microglial proinflammatory responses, thereby mitigating neuroinflammation within the NAc and lessening depressive symptoms through the Akt signaling pathwa [129]. NE is fundamental in regulating sleep, emotions, and attention in humans. As a precursor to NE, DA's involvement is crucial, and the therapeutic use of 5-HT and NE reuptake inhibitors (SNRIs) has been shown to effectively alleviate symptoms associated with PSD [125]. This highlights the intricate interplay between the inflammatory response, neurotransmitter systems and the development of depressive symptoms following a stroke, underscoring the potential of targeted neurotransmitter system interventions in managing PSD.

Amino acid neurotransmitters, specifically the excitatory glutamate (Glu) and the inhibitory GABA, play pivotal roles in maintaining neural equilibrium. Disruption in the balance between these neurotransmitters is linked to the development of PSD. Within the neural circuitry, glutamate neurons, adjacent astrocytes and GABAergic neurons form excitatory/inhibitory functional units essential for neuro-nutrition, neural plasticity and cognitive functions such as memory [130]. Glutamate, essential for neural communication, can also act as a potential neurotoxin. Inflammatory cytokines accelerate Trp degradation, leading to an increase in QA within glial cells, a neuroactive metabolite known for its excitotoxicity through N-methyl-D-aspartate (NMDA) ionotropic glutamate receptors. This process results in an excessive glutamate release, leading to an accumulation in the synaptic cleft. The accumulation can cause excitotoxicity, trigger apoptosis, diminish neural plasticity and ultimately lead to neuronal loss, thereby contributing to the onset of PSD [131]. Ketamine, a non-competitive N-methyl-D-aspartate receptor (NMDAR) antagonist, targets the GluN2B subunit of NMDARs on GABAergic neurons. It enhances glutamatergic activity and could be an effective treatment for PSD [132]. Similarly, blood glutamate scavengers like oxaloacetate and α-ketoglutarate have demonstrated potential in alleviating PSD symptoms in preclinical studies [133]. Transcranial magnetic stimulation therapy (TMS) has been observed to enhance the synthesis and release of 5-HT, DA, and NE, as well as modulate inhibitory/excitatory amino acid neurotransmitters. It also mitigates inflammatory responses, offering relief from PSD [134].

### 2.5 Brain-Derived Neurotrophic Factor

Brain-derived neurotrophic factor (BDNF), a member of the neurotrophin family, is a crucial neurotrophic factor, particularly vital for neural repair and regeneration in the hippocampus and cortex [135]. Current research on BDNF's role in PSD primarily focuses on its influence during synthesis and maturation. Notably, the balance between proBDNF and mature BDNF is believed to play a key role in the development of PSD [136]. The formation of functionally active subtypes such as proBDNF and mBDNF is one of the most important characteristics of BDNF synthesis [137]. These subtypes are not only essential for precise cellular control but also necessary for maintaining physiological homeostasis. Particularly in the hippocampal region, reduced BDNF expression impacts neuronal plasticity as well as the structure and function of synapses [138]. Studies indicate that low BDNF levels in the hippocampus are associated with reduced neurogenesis, potentially leading to impaired PSD development [139, 140].

BDNF plays a key role in modulating depressive symptoms in brain regions such as the prefrontal cortex, hippocampus and amygdala, with its high expression in the hippocampus being closely linked to neuronal plasticity and synaptic function. Research by Esalatmanesh et al. has highlighted the significant role of BDNF in hippocampal synaptic function [141]. In Alzheimer's Disease (AD) patients, the olfactory system, including the hippocampal CA1 area, exhibits an early activation of the endoplasmic reticulum stress response, indicating a high sensitivity of the hippocampal CA1 area and the entorhinal cortex in responding to neurodegenerative changes [142]. Among BDNF subtypes, mBDNF and proBDNF interact with different receptors, TrkB and p75 neurotrophin receptor (p75NTR), promoting and inhibiting neural plasticity respectively. Activation of TrkB is associated with anti-depressive inflammatory responses [143, 144], while p75NTR is involved in various nonspecific neurotrophic [145]. Studies have shown that proBDNF and mBDNF share analogous biological activities. Opreating as a functional protein, proBDNF binds to the low-affinity nerve growth factor receptor p75NTR, further activating downstream signals such as Rho family member A (RhoA) and c-Jun N-terminal kinase (JNK), thereby inducing apoptosis and thus triggering PSD [146]. The expression of proBDNF and mBDNF shows dynamic changes during different stages of brain development [147]. In a rat model of PSD, serum levels of proBDNF are significantly increased in PSD rats, with a marked decrease in the ratio of mBDNF to proBDNF. The increase in proBDNF and the decrease in mBDNF are related and this imbalance may lead to a decline in hippocampal neural functions, thereby promoting the development of PSD [148].

In a study, the administration of exogenous BDNF to the hippocampal region of rats exhibiting depression-like behavior induced by chronic unpredictable mild stress (CUMS) was observed to ameliorate depressive symptoms. Conversely, the introduction of exogenous proBDNF was found to exacerbated depression-like behavior [149]. proBDNF, encoded by BDNF mRNA, is typically converted into mBDNF through proteolytic cleavage. In PSD models [150, 151], the hydrolysis process of proBDNF is inhibited, leading to elevated levels of proBDNF in rats. Recent studies have shown that antidepressants like Pimavanserin significantly increase mBDNF levels in rat plasma, providing new insights into changes in BDNF levels [152]. Moreover, tPA plays a more complex role in the functional regulation of BDNF. Research by Haidar et al. identified significant differences in the expression levels of mBDNF and its precursor proBDNF in the rat brainstem region (RVLM) under various activity states. These differences may be associated with changes in tPA expression levels, consequently impacting the maturation and function of BDNF [153]. Furthermore, a study by Hao Dong et al. indicated that electroacupuncture treatment could improve depression-like behavior in post-stroke rats by activating the tPA/BDNF/TrkB signaling pathway [148]. These findings suggest that the levels of proBDNF and mBDNF in the hippocampal region along with their mechanisms of action, differ. The balance between them may impact the functional activity of BDNF, and intervening in their ratio could promote hippocampal neuronal regeneration, improve depressive symptoms, and offer new research directions for treating PSD.

In the hippocampal CA1 area, proBDNF and mBDNF exhibit distinct functions. proBDNF, through binding to the p75NTR receptor, induces long-term depression (LTD) of synaptic efficacy, thereby affecting synaptic plasticity. In contrast, mBDNF, binding to the TrkB receptor, promotes long-term potentiation (LTP) of synaptic efficacy, contributing to synaptic stability and enhancement. Recent research has found that Arc gene expression plays an important role in regulating LTP and synaptic plasticity in the hippocampal CA1 area [154]. Recent studies have shown that the binding of proBDNF to p75NTR not only activates JNK and RhoA signaling pathways but may also affect NF-κB activation. Research by Yang et al. indicates that in a PSD model, the expression of proBDNF increases, and its binding to p75NTR activates apoptosis-related proteins through the RhoA-JNK signaling pathway, promoting the development of PSD [155]. Another study found that Dexmedetomidine could alleviate neurotoxicity in the developing brain caused by Sevoflurane by regulating the proBDNF-p75NTR-RhoA pathway and the BDNF-TrkB-CREB pathway [156]. Latest research by Yang et al. has revealed that in patients with Major Depressive Disorder (MDD), there is an elevation in the expression of proBDNF and its receptors in peripheral blood mononuclear cells. This is associated with inflammatory markers, suggesting a potential link between proBDNF/p75NTR signaling and NF-κB activation [157]. This modulates subsequently the expression of inflammatory factors and related apoptotic proteins PSD95, synaptophysin (SNP) and P-cofilin or inhibits the expression of anti-apoptotic proteins like RIP2, cleaved caspase-3 and cytochrome C to promote PSD. Studies show that in PSD rats, proBDNF binds to the p75NTR receptor and activates the RhoA/JNK signaling pathway, promoting apoptosis and inhibiting synaptic regeneration [155], which might contribute to the development of PSD.

The activation of the JNK signaling pathway is crucial for the functionality of NF-κB, as it facilitates the entry of NF-κB into the nucleus and activates the transcription of inflammatory genes. This process leads to the release of more inflammatory factors, creating a vicious cycle that exacerbates inflammation and may trigger the development of PSD [158]. NF-κB, a key transcription factor, in conjunction with the NLRP3 inflammasome to drives inflammatory responses. This collaboration induces the expression of inflammatory mediators such as IL-1β, IL-6 and TNF-α as well as upregulating the synthesis of NLRP3, contributing to various inflammatory, autoimmune, and neurological diseases [159]. These inflammatory pathways play a significant role in disorders such as depression, AD and Parkinson's disease, making them key therapeutic targets in the inflammatory response of PSD [160]. Additionally, other signaling pathways like cAMP/protein kinase A (PKA) may also play a role in regulating NF-κB and NLRP3-related inflammatory responses [161], providing new research directions for the treatment of PSD.

Recent studies have further revealed how inflammatory cytokines influence the BDNF-TrkB-CREB signaling pathway through the modulation of serotonin neurotransmission. These studies underscore the importance of the 5-HT1A and 5-HT2A receptors in this process. Research by Cao et al. demonstrated that the traditional Chinese medicine formula 'Si Ni San' improves depression-like behavior in rats subjected to maternal separation by activating the 5-HT1A receptor/CREB/BDNF pathway. This finding emphasizes the role of the 5-HT system and its receptors in modulating the BDNF-TrkB-CREB signaling pathway [162]. Furthermore, recent research by Shimada et al. showed that isovaleraldehyde upregulates BDNF and CREB phosphorylation via the PKA pathway, exhibiting neuroprotective effects. This indicates that PKA activation and subsequent CREB phosphorylation play a central role in regulating BDNF expression and the process of depression, emphasizing the significance of the PKA-CREB-BDNF signaling pathway in neuroprotection and antidepressant effects [163]. Studies have shown that inhibiting CREB signaling in the hippocampus disrupts the expression of genes including BDNF, thereby inducing depression-like behavior [164]. BDNF coupled with p-CREB plays a key role in neurogenesis and emotional regulation, forming a fundamental mechanism for functional recovery and neural plasticity [165, 166]. Activation of PKA enhances CREB phosphorylation, thereby regulating the transcription of various genes including BDNF [167]. In PSD model mice, the protein levels of BDNF and p-CREB in the hippocampus are reduced, while mice receiving antidepressant treatments like fluoxetine show significantly increased expression of BDNF and p-CREB in the CA1 area [168]. This suggests that the roles of BDNF and p-CREB in cell proliferation, apoptosis, synaptic genesis and memory formation may be closely linked to the development of PSD. Latest research by Liu et al. revealed that ARN-3236, a selective SIK2 inhibitor, produces strong antidepressant-like effects through the hippocampal CRTC1-CREB-BDNF pathway, offering new insights into understanding the roles of BDNF and p-CREB in neurogenesis, emotional regulation and the development of PSD [169].

BDNF plays a crucial role in the pathophysiology of PSD, primarily through its interaction with TrkB. This interaction triggers autophosphorylation of tyrosine residues and receptor dimerization, activating several downstream signaling pathways including PI3K/AKt pathway, MAPK pathway, and phospholipase C (PLC-Y)/protein kinase C (PKC) pathway. These pathways collectively enhance synaptic plasticity, promote neuronal growth and survival, thereby protecting and nourishing the nervous system [170]. Conversely, TrkB lacking the tyrosine kinase domain negatively impacts PSD [171]. When TrkB lacks autophosphorylation, it binds and internalizes BDNF, leading to reduced cell proliferation, increased apoptosis, and impairments in synaptic genesis and memory formation in the hippocampal region, thereby promoting the development of PSD. Recent research by Xu et al. shows that Huang-Qi-Glycoside alleviates depressive-like phenotypes by activating the BDNF-TrkB-mTORC1 signaling pathway and enhancing synaptic plasticity, offering new insights into the neuroprotective roles of BDNF and TrkB in PSD and their impact on synaptic plasticity [172].

**Figure 4.**
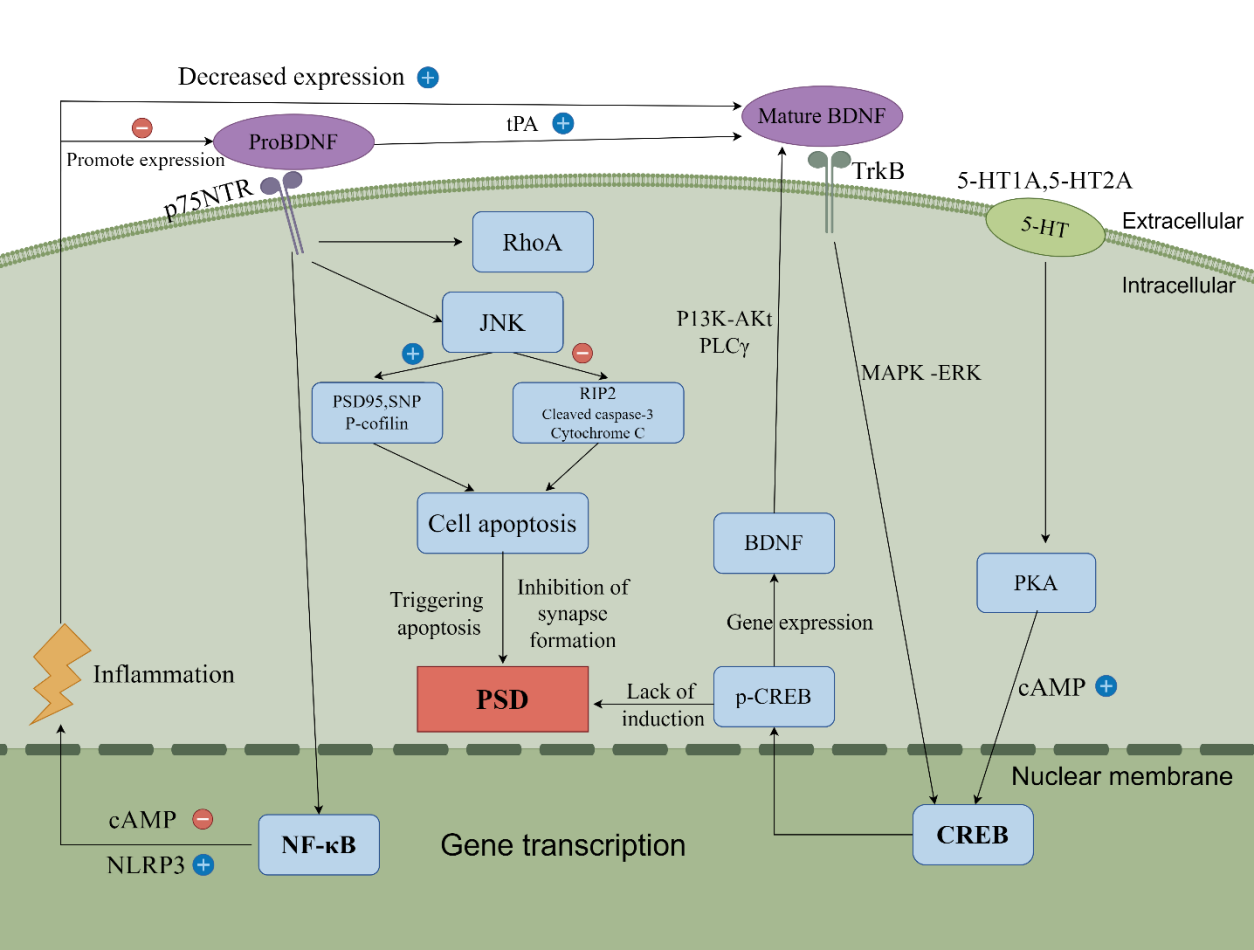
**Brain derived neurotrophic factors and post-stroke depression inflammation**. This figure illustrates the roles of BDNF and its isoforms, mBDNF and proBDNF, in hippocampal neuroplasticity, highlighting the promotion of neuronal regeneration by mBDNF/TrkB via the CREB pathway, and the modulation of cell apoptosis by proBDNF/p75NTR through the NF-κB and JNK pathways. Additionally, the figure briefly describes the regulatory effects of 5-HT1A and 5-HT2A receptors on BDNF expression and emotional response, as well as the involvement of inflammatory factors in the development of PSD by modulating these signaling pathways (by Figdraw).

In summary, neurotrophic proteins can activate different cellular mechanisms depending on the type of receptor they interact with. In vivo studies have confirmed the impact of inflammation on BDNF expression in the brain and further explored the influence of pro-inflammatory cytokines on BDNF gene and protein expression in PSD. Research by Benjamin Morris assessed inflammatory predictors of depressive symptoms, particularly in the context of exercise interventions. The study explored the correlation between pro-inflammatory cytokines such as TNFα, IL-1β and IL-6 with hippocampal volume and scores on the Beck Depression Inventory [173]. This suggests that the dysregulation of the interaction between BDNF and its receptor TrkB may not only be an independent pathological factor but also a disruption in neurotrophic factor signal transduction. Such disruption may increase the brain's vulnerability to subsequent damage, thereby promoting the development of PSD. (Shown in Fig. 4).

BDNF plays a key role in the development of PSD, particularly in terms of the changes in its expression in the hippocampal and prefrontal cortex areas. However, Shan et al. noted that there is inconsistency in the findings regarding the relationship between BDNF levels and PSD [174]. While some studies have shown reduced BDNF levels in PSD patients, others have not found this correlation, which may be attributed to differences in study design, sample selection, disease stage or measurement methods. Furthermore, mBDNF and proBDNF have opposing roles in neural plasticity and apoptosis. This further indicates that, although BDNF is considered to play a significant role in PSD, its exact mechanisms of action remain unclear, suggesting that BDNF might influence PSD through more complex or currently not fully understood mechanisms.

### 2.6 Dysregulation of hypothalamic-pituitary-adrenal (HPA) axis

The hypothalamic-pituitary-adrenal (HPA) axis is a fundamental component of the body's neuroendocrine system, acting as a self-regulating mechanism for stress responses. It facilitates communication between the CNS and peripheral organs, playing a pivotal role in maintaining internal balance and responding to stress [175]. The axis initiates its response in the hypothalamus, where the paraventricular nucleus synthesizes and releases corticotropin-releasing hormone (CRH), stimulating the production and release of adrenocorticotropic hormone (ACTH) and glucocorticoids (GCs). These GCs, in turn, exert a negative feedback effect on the hypothalamus and pituitary gland, regulating the system's activity.

Stroke events can significantly activate the HPA axis by causing neuronal damage, which acts as a stressor. This activation is further exacerbated by cytokines released from the injured neurons, with pro-inflammatory cytokines such as IL-1, IL-6, CRP, TNF-α and IFN-γ being potent stimulators of the HPA axis [176]. This results in an increased secretion of CRH and ACTH, leading to a disruption in the negative feedback loop and sustained HPA axis dysfunction.

The stroke-induced neuronal damage prompts an upsurge in CRH and ACTH levels, leading to the suppression and diminished functionality of glucocorticoid receptors. This disruption interrupts the negative feedback loop of the HPA axis, perpetuating its dysfunction. Pro-inflammatory cytokines can upregulate the expression of IDO, and increased GC levels enhance the activity of tryptophan-2,3-dioxygenase (TDO) [177].

**Figure 5.**
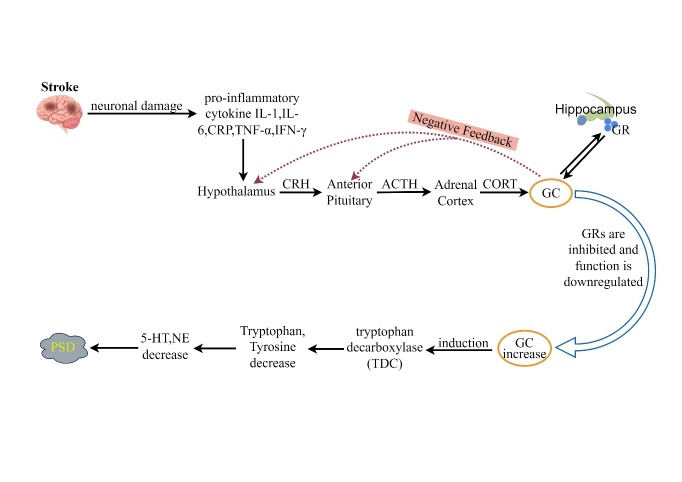
**Dysregulation of HPA axis**. Following a stroke, neurons sustain damage and release pro-inflammatory cytokines such as IL-1, IL-6, CRP, TNF-α, and IFN-γ. These cytokines can act on the HPA axis, triggering positive feedback regulation. The GCs produced by the HPA axis can, in turn, act on the HPA axis to form a negative feedback mechanism. The HPA axis maintains its normal function through these positive and negative feedback regulatory mechanisms. The hippocampus serves as the central hub for the negative feedback regulation of the HPA axis. Glucocorticoid receptors in the hippocampus can bind with GCs from the HPA axis, participating in the negative feedback regulation of the HPA axis. If glucocorticoid receptors are inhibited or their function is reduced, glucocorticoid levels may rise, thereby inducing the expression of tryptophan decarboxylase, degrading tryptophan and tyrosine, reducing the synthesis of 5-HT and NE, and potentially leading to the development of PSD (by FIgdraw).

Furthermore, elevated glucocorticoid levels in the plasma can trigger the upregulation of Trp decarboxylase, resulting in the depletion of the precursors for 5-HT and NE, thereby reducing their synthesis and contributing to the development of PSD [178]. The hippocampus, which typically regulates the HPA axis, may become impaired, leading to excessive cortisol levels that may inflict direct neuronal damage or hippocampal injury, exacerbating depressive symptoms. The overactivity of the HPA axis in PSD is marked by increased levels of CRH, ACTH and GC. Treatment with antidepressants like sertraline and escitalopram has been shown to mitigate HPA axis dysfunction, thereby enhancing neural function recovery and alleviating depressive symptoms [178, 179] (Shown in Fig. 5). The effectiveness of these antidepressant medications in modulating the HPA axis function correlates with symptom severity, though it remains unclear whether these improvements directly result from the alleviation of HPA axis overactivity or from the antidepressants' effects on PSD [178]. CRH, a crucial regulator of BDNF, can influence CREB phosphorylation when overexpressed in the hippocampus, leading to reduced BDNF transcription in the CA1 area [180]. Hence, targeting the HPA axis for modulation emerges as a promising therapeutic approach for addressing PSD.

### 2.7 Inflammatory pathways and oxidative stress-induced neuronal damage

Oxidative stress emerges as a pivotal element in stroke pathophysiology, especially during cerebral ischemia-reperfusion phases. This stage is characterized by alterations in mitochondrial dynamics and damage to the mitochondrial inner membrane, resulting in mitochondrial dysfunction and a surge in ROS production. Post-stroke, there is a marked increase in dynamin-related protein 1 (Drp1), which not only triggers apoptosis but also intensifies mitochondrial dysfunction by elevating ROS levels, thereby hindering the glutathione-dependent antioxidant defense system [181]. Additionally, ROS generation by nicotinamide adenine dinucleotide phosphate oxidase 2 (NOX2) exacerbates acute brain injury and might also facilitate brain function recovery [182].

The Thioredoxin-interacting protein (TXNIP), an inherent regulator of the Thioredoxin (TRX) system, associates with the NLRP3 inflammasome following a stroke, instigating an inflammatory cascade [183]. NLRP3 activation plays a pivotal role in ischemic stroke evolution, where mitigating the TXNIP/NLRP3 pathway can diminish oxidative stress and inflammation, thus offering neuroprotection [184]. The interface between mitochondria and the endoplasmic reticulum, known as mitochondria-associated endoplasmic reticulum membranes (MAMs), is instrumental in inflammasome assembly, facilitating ROS production amidst mitochondrial dysfunction and activating the NLRP3 inflammasome [185]. After a stroke, mitochondria govern the NLRP3 inflammasome by discharging molecular contents, which release pro-inflammatory cytokines such as IL-1β and IL-18, driving the inflammatory response, potentially linked to post-stroke depressive-like behaviors.

Oxidative stress is implicated in depression onset, with stroke-induced oxidative stress elevating ROS levels, lipid peroxidation, protein oxidation and DNA damage within neural tissues, thereby contributing to PSD development [186]. PSD symptoms are characterized by systemic immune inflammation and oxidative stress post-stroke, inducing neuroemotional toxicity and affecting gray and white matter plasticity, manifesting in depressive symptoms [187]. Moreover, oxidative stress escalates plasma levels of specific metabolites like phenylalanine, tyrosine, 1-methylhistidine, 3-methylhistidine and LDLC3-(CH2)N-, closely linked to PSD [188].

**Figure 6.**
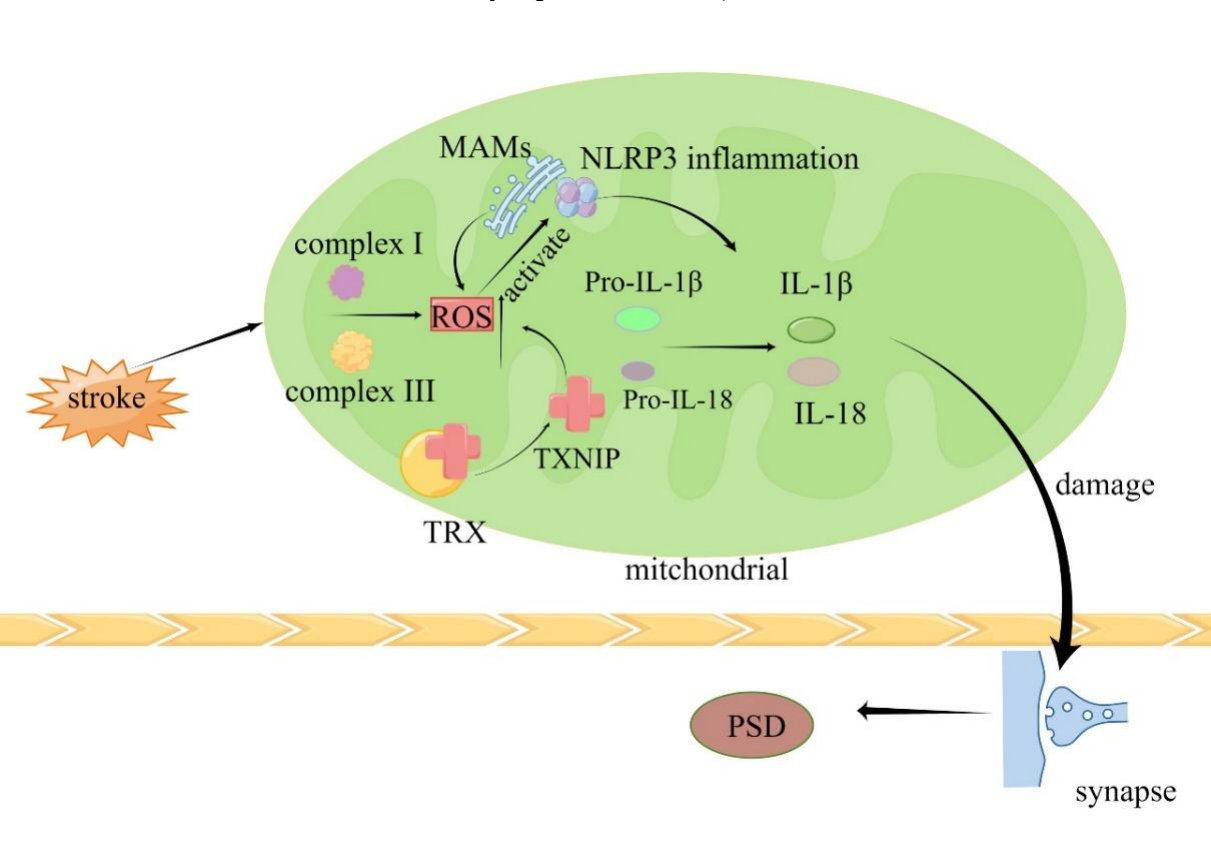
**Inflammatory pathways and oxidative stress-induced neuronal damage**. After the onset of stroke, ROS produced by mitochondrial complexes I and III and TXNIP dissociated from the TRX complex promote ROS production, activating the resting NLRP 3 inflammasome on the endoplasmic reticulum, and the inflammasome is activated for lysis into IL-1 β precursor and IL-18 precursor, producing IL-1 β and IL-18. Proinflammatory factors damage synapses leading to PSD (by Figdraw).

Preservation of mitochondrial functionality is thus pivotal for neuronal and overall nervous system health. The transplantation of external mitochondria to mitigate mitochondria-induced ROS and modulate synaptic marker expression can ameliorate post-stroke cognitive deficits [189]. Recent investigations highlight that multifunctional nanoparticles designed for ROS-responsiveness and mitochondrial targeting can precisely deliver and control drug release within the ischemic penumbra. Delivered intranasally to circumvent the BBB, these nanoparticles directly reach the ischemic penumbra, exerting cellular antioxidant, mitochondrial targeting and neuroprotective effects [190]. ROS, a central instigator of irreversible ischemic region damage, triggers not only an inflammatory response in brain tissues but also thrombus formation. Hence, a synergistic approach focusing on ROS clearance, thrombosis inhibition and BBB permeability enhancement can substantially improve the post-stroke microenvironment and safeguard brain tissue [191]. (The related mechanisms are illustrated in Fig. 6).

## 3. Targeted Therapies for Inflammation in PSD

### 3.1 Anti-inflammatory agents

#### 3.1.1 Non-steroidal anti-inflammatory drugs (NSAIDs)

In the exploration of targeted treatment strategies for inflammation in PSD, the role of NSAIDs has garnered considerable attention. As one of the common complications of stroke, the pathogenesis of PSD is closely linked to neuroinflammation. Recent studies have highlighted the clinical efficacy of various treatment approaches including selective serotonin reuptake inhibitors (SSRIs), NE and specific serotonin antidepressants (NaSSAs), anti-inflammatory drugs and vitamin D [192]. Particularly, anti-inflammatory drugs play a significant role in regulating neuroinflammation and improving symptoms of PSD.

NSAIDs function by inhibiting cyclooxygenase enzymes [193], among which cyclooxygenase-1 selective inhibitors, primarily represented by aspirin, are classical NSAIDs with broad-spectrum action and multiple action sites. Aspirin is a suitable medication for early prevention and treatment during the acute phase of stroke and in the early stages of PSD. Basic research has shown that the combination of aspirin with clopidogrel can alleviate the inflammatory response in ischemic stroke by inhibiting the NF-kB/NLRP3 pathway [194]. The 2018 guidelines released by the American Heart Association and the Stroke Association recommend the combined use of aspirin and clopidogrel within 24 hours after a mild stroke or transient ischemic attack, continuing for three weeks, to help prevent early stroke recurrence within three months from symptom onset [195]. Reducing the inflammatory response and preventing stroke recurrence plays a positive role in the occurrence and development of PSD.

Neuroinflammation occupies a central position in the pathogenesis of PSD. Inflammatory cytokines such as TNF-α and interleukin-1β (IL-1β) are upregulated in PSD. These cytokines activate inflammatory pathways in the hippocampal and frontal cortex areas, leading to neurodegenerative changes and impaired neural conduction. Studies have shown that anti-inflammatory treatment, particularly the use of NSAIDs, can alleviate the symptoms of PSD by inhibiting these inflammatory pathways [196].

#### 3.1.2 Immune-modulating agents

Immunosuppressants are commonly used in clinical practice to modulate the body's immune response and correct immune dysfunctions for therapeutic goals. They have shown effectiveness in managing autoimmune diseases and organ transplantation. Research indicates that thymosin-alpha 1, a crucial chemotactic factor for leukocyte infiltration in brain tissue, can regulate the permeability of the BBB when combined with CCD2. This combination inhibits cerebral edema, reduces leukocyte infiltration and the expression of inflammatory mediators [197]. The use of immunosuppressants has significantly improved the cure rate of stroke-related infections, enhancing clinical outcomes, promoting recovery from stroke, shortening the duration of inflammation resolution and reducing the accumulation of inflammatory cells within the skull [198]. During treatment, immunomodulatory therapy effectively improves fibrinogen levels, NIHSS scores and more, demonstrating considerable therapeutic value in the clinical treatment of PSD.

In summary, NSAIDs demonstrate significant potential in the targeted treatment of inflammation associated with PSD. By modulating the inflammatory response, they contribute to alleviating the symptoms of PSD and improving cognitive functions. However, the long-term effects and potential side effects of anti-inflammatory treatments still require further research to ensure their safe and effective clinical application. Future studies may focus on exploring more precise anti-inflammatory treatment targets and developing safer and more effective medications to better address the complex clinical challenge of PSD.

### 3.2 Antioxidant therapies

PSD is not only related to neurotransmitter imbalances but is also closely linked to oxidative stress and neuroinflammation. Atherosclerosis, as the pathological basis of stroke, leads to the production of various oxidative products and inflammatory factors post-ischemia reperfusion, damaging the BBB and playing a crucial role in the onset and progression of PSD.

Atherosclerosis is the pathological foundation of stroke. From the entire process of atherosclerosis to the damage of the BBB by various oxidative products and inflammatory factors post-ischemia reperfusion, the roles of oxidative stress and inflammatory responses persist throughout PSD. Anti-inflammatory and antioxidant treatments can target the underlying causes of PSD. Antioxidant drugs are mainly divided into two categories. First, natural antioxidants such as vitamin E, vitamin C, catalase and glutathione [199], which neutralize free radicals and prevent the accumulation of excessive ROS in the body. Second, synthetic antioxidants, which can be obtained from external food or supplements. Two are both effectively preventing oxidative damage caused by ROS. Besides individual medication, considering the heterogeneity of stroke etiology and the involvement of various harmful mechanisms in post-stroke injury, the combined use of antioxidants with other treatment strategies is more advantageous. Recent research by Kaur et al. shows that melatonin has a modulatory effect on pain behaviors in animal models of central post-stroke pain, possibly by alleviating oxidative stress and neuroinflammatory responses [200]. This study highlights the role of oxidative stress in post-stroke pain and depressive symptoms, offering a new perspective for antioxidant therapy.

### 3.3 Neurotrophic factors and neurogenesis enhancers

BDNF can protect damaged brain tissue through multiple pathways and promote the recovery of brain cells after ischemia, while reducing the spread of the ischemic penumbra in cerebral infarction and lowering the incidence of PSD. Recent studies, such as that by Liu et al., have found that Aloe-emodin (AE) shows potential therapeutic effects in a rat model of PSD [201].AE improves depressive symptoms by increasing the expression of BDNF and neurotrophic factor 3 (NTF3), increasing the number of neurons, and reducing brain tissue damage. Additionally, AE can regulate the expression of aquaporins, such as (aquaporins; AQPs) AQP3, AQP4, AQP5, aiding in maintaining the homeostasis of the nervous system and alleviating cerebral edema. These findings suggest that AE could be an effective potential solution for treating PSD.

On the other hand, the role of neurotrophic factors in regulating hippocampal synaptic plasticity should not be overlooked. The hippocampal region, crucial for regulating emotional and cognitive functions, plays a vital role in the treatment of PSD, particularly the action of neurotrophic factors like BDNF in this area. Latest research indicates that neurotrophic factors can influence depressive behaviors by modulating hippocampal synaptic plasticity, offering a new perspective for a better understanding of the pathology of PSD and improving its treatment methods [202, 203].

Overall, the application of neurotrophic factors in the treatment of PSD holds broad prospects. Future research might continue to explore different types of neurotrophic factors and how they can be used in conjunction with other treatments such as antidepressants, anti-inflammatory drugs to provide more comprehensive treatment strategies.

### 3.4 Psychiatric interventions combined with anti-inflammatory strategies

PSD exhibits distinct depressive manifestations and as one of the most prevalent mental disorders, depression has been associated with the inflammatory hypothesis since the last century [204]. Depressive disorders are linked to chronic, low-level inflammatory responses, cell-mediated immunity and the activation of compensatory anti-inflammatory reflex systems [205]. A meta-analysis showed that high levels of CRP and IL-6 are associated with future depressive symptoms [206], suggesting that inflammatory cytokines are potentially important biological markers in depression research. Numerous clinical studies indicate that adjunctive anti-inflammatory drugs can enhance the therapeutic effect of antidepressants [207]. Thus, combining these treatments is a clinically appropriate strategy.

Recent research points out that anti-inflammatory drugs, acetylcholinesterase inhibitors, and selective serotonin reuptake inhibitors play a role in treating PSD, but their efficacy requires further investigation and validation [208]. Minocycline, as an antibiotic with anti-inflammatory, antioxidative and anti-apoptotic properties, is increasingly receiving attention for its application in psychiatric and neurological disorders. Systematic reviews and meta-analyses suggest that minocycline may have potential benefits in treating PSD, especially when combined with psychiatric interventions and anti-inflammatory strategies. These studies emphasize the role of minocycline in regulating neuroinflammation, oxidative stress and neuroprotection, offering new pharmacological options for PSD treatment [209].

Synthesizing these studies, we can see the significance of psychiatric interventions and anti-inflammatory strategies in the treatment of PSD. This integrated treatment approach can target the multifaceted pathophysiological mechanisms of PSD, thereby offering more comprehensive therapeutic effects. Psychological interventions can help patients cope with emotional distress and social challenges, while anti-inflammatory drugs and other medical treatments directly act on the neurobiological level, improving neuroinflammation and oxidative stress and promoting the repair and regeneration of neural cells.

## 4. Genetic and Epigenetic Factors

The pathogenesis of PSD involves complex factors, with genetic variations considered to be related to its occurrence. In comparison to studies using animal models, the expression of some common genes in human major depressive disorder, such as Htr2a, Ntrk3, Crhr1, Ntrk2, Crh, complement component 3 and fatty acid binding protein 7 undergoes changes in both short-term and long-term treatments in depression animal models [210]. Additionally, individuals carrying polymorphisms in the serotonin transporter protein promoter or the STin2 genotype have been found to be more prone to PSD. Some studies further suggest an association between the s/s variant in the serotonin transporter gene promoter region and post-stroke mood instability [211]. Moreover, the presence of a 5-HTTLPR gene mutation has been found to significantly elevate the risk of developing PSD, highlighting the critical role of genetic factors in individual susceptibility to depression post-stroke [212].

In the realm of PSD research, initial investigations with a limited cohort of Caucasian participants have revealed that variations in the 5-HTTLPR gene might influence the genetic predisposition to severe depressive episodes following a stroke [213]. Notably, the presence of the S allele has been correlated with a heightened risk of PSD. This allele is theorized to diminish serotonin production, which in turn can lead to a decrease in the reuptake and storage of this crucial neurotransmitter, potentially contributing to the development of PSD [214]. Further research conducted by Fang J and colleagues supports this hypothesis, showing a higher prevalence of the S/S homozygous genotype within the PSD-afflicted demographic, thereby reinforcing the link between the S allele and an increased likelihood of PSD [215]. In contrast, other genetic variations within the 5-HTTLPR gene appear not to have a significant correlation with PSD, adding layers of complexity to the genetic underpinnings of this condition. Intriguingly, some research posits that the G allele might serve as a predictive marker for depression due to its potential role in downregulating the serotonin transporter, further complicating the genetic landscape of PSD [216]. These insights underscore the intricate genetic factors contributing to PSD and highlight the need for further, more expansive research to unravel these complex genetic relationships fully.

Regarding the LG genotype, emerging research posits that its impact parallels that of the S allele, linking it to low expression variants and a predisposition to PSD [217]. These findings underscore the intricate interplay between genetic variances and PSD. Despite initial insights, the constraints of small participant pools, intricate genetic interrelations, and the interplay of additional genetic and environmental factors warrant expansive future investigations to corroborate these preliminary results.

In the domain of epigenetics, the methylation status of the BDNF promoter region is pivotal, as it can modulate BDNF release, subsequently influencing depression risk [218]. A longitudinal analysis by Jae-Min Ki et al. highlighted a direct correlation between elevated BDNF methylation percentages and PSD incidence, suggesting that heightened methylation might suppress BDNF secretion post-stroke, thereby elevating depression risk. Given BDNF's crucial role in the survival of diverse CNS structures, enhanced methylation could impede neuronal repair in stroke-impacted brain regions, heightening depression susceptibility. This study, however, didn't find a significant interplay between methylation levels and genotypic variations [219],diverging from prior studies that linked methylation status to genotypic dependencies [220]. This discrepancy underlines the necessity for further inquiry into BDNF's role in PSD [221].

Additionally, the intersection of epigenetics and epitranscriptomics with the gut microbiota offers intriguing prospects for understanding and addressing PSD, suggesting that these mechanisms could pave the way for innovative therapeutic strategies. Nonetheless, a comprehensive grasp of these interactions remains an area ripe for in-depth research [222].

Gender significantly influences the etiology and severity of many diseases, with depression being notably prevalent. Women are more susceptible to depression, particularly severe forms, a disparity attributed to various factors including gender-specific gene expression related to depression, leading to distinct emotional and behavioral manifestations. For instance, increased expression of serotonin and glutamate receptor genes has been observed in females, whereas males show a reduction in BDNF-dependent genes and BDNF receptor TrkB expression post-depression [223-225]. Gender also critically impacts the pathological processes post-stroke, highlighting the necessity of considering gender differences in stroke and depression research.

A deeper exploration into the gender-specific epigenetic and transcriptomic alterations post-stroke, and the resultant changes in depression-related transcriptional products, is imperative. Research has already identified gender variations in TET3 activity within the ischemic brain [226]. Furthermore, the gut microbiota composition in both animals and humans displays gender dependencies [227-230],suggesting that these differences could significantly contribute to epigenetic and transcriptomic variations. This gender-specific perspective in microbiota composition offers a more holistic, interdisciplinary approach to investigating stroke and depression.

These insights into gender differences underscore the complexity of PSD pathogenesis and hint at the potential for tailored treatment strategies, acknowledging the intricate interplay of genetic, epigenetic, and environmental factors influenced by gender.

## 5. Non-inflammatory factors

PSD arises from a constellation of factors, with this investigation predominantly delving into the inflammatory mechanisms contributing to PSD episodes. Nonetheless, akin to the multifactorial nature of psychiatric disorders, non-inflammatory contributors such as psychological factors, personality traits, social determinants and genetic predispositions also merit consideration in the context of PSD. The likelihood of PSD is heightened in patients who are female, have a psychiatric history, have undergone extensive or multiple strokes, have sustained damage to the frontal lobes or basal ganglia, have experienced a stroke within the last year, lack social support or have significant disabilities, yet these associations are not definitive [231]. Evidence advocates for conceptualizing PSD as a biopsychosocial disorder, intricately shaped by a myriad of etiological factors including psychological states, social support, lifestyle choices, and genetic makeup. These elements potentially exert a pronounced influence on specific manifestations or symptoms of PSD, thereby impacting its treatment and onset [232]. Personality traits and adverse psychological states also emerge as pivotal predictors of PSD. Prospective research has corroborated the association between Type D personality and an elevated risk of PSD, with such personality independently prognosticating PSD at three months post-stroke. Moreover, the interplay between negative affectivity (NA) and social inhibition (SI) within the Type D personality framework significantly correlates with PSD, underscoring the interrelation among personality traits, negative emotional states, social reticence and PSD onset [233]. Contradictory evidence persists regarding the role of social support in PSD; while the extent of social connections inversely relates to PSD severity, the correlations between living conditions, marital status, and PSD remain inconsistent [234]. Earlier findings indicated that a paucity of social support at admission was linked to PSD onset at a three-month follow-up [235]. Furthermore, the risk of PSD development is associated with patient age, with advancing age exacerbating immune system dysregulation and accelerating neurodegenerative processes that are pivotal in stroke-related outcomes [1]. Research indicates that individuals aged 55-64 exhibit a nearly threefold increase in PSD risk [236]. The author posits that future investigations should broaden their scope to encompass a more extensive array of non-inflammatory factors in elucidating PSD mechanisms.

## 6. Challenges and future directions

This review encapsulates the findings from animal experiments and clinical investigations pertaining to PSD, shedding light on the complexities of its pathogenesis and its implications for future diagnostic and therapeutic strategies. Presently, the diagnostic framework for PSD remains nebulous, devoid of definitive guidelines. The quest for predictive markers of PSD encounters two primary obstacles. First, the clinical methods for diagnosing PSD lack specificity and cannot demonstrate a direct correlation with PSD [237]. Second, the effectiveness of preventive antidepressant interventions in reducing the incidence of PSD has not been proven [238]. Initial manifestations of PSD may elude detection in some patients, with post-stroke cerebral damage precipitating inflammatory responses that, albeit significant, do not serve as reliable PSD predictors, necessitating the identification of more precise biomarkers [239]. For instance, elevated CRP levels during stroke's acute phase hint at an augmented PSD risk [112], however, this does not directly delineate the specific interplay between CRP levels and PSD. Current diagnostic practices lean heavily on subjective instruments like the Hamilton Depression Rating Scale (HDRS) and the Patient Health Questionnaire-9 (PHQ-9), with a glaring absence of objective biomarkers. Imaging modalities, notably magnetic resonance imaging (MRI), emerge as pivotal in PSD diagnostics, with acute post-stroke MRI revealing characteristic ipsilateral diffusion alterations and cerebral edema [240]. Functional MRI illustrating diminished excitability in lesion-impacted cerebral regions among PSD sufferers [241]. Observations of white matter microstructural anomalies in the frontal and temporal lobes and the corpus callosum in PSD patients underscore the potential of early neuroimaging in gauging PSD risk [242]. Moreover, standardized psychiatric and neurological evaluation tools, including the Mini International Neuropsychiatric Interview-Plus (MINI-Plus), the Hospital Anxiety and Depression Scale (HADS), and the National Institutes of Health Stroke Scale (NIHSS), contribute to PSD assessment [243]. Nevertheless, the clinical diagnosis of PSD predominantly aligns with the depressive disorder criteria outlined in the Diagnostic and Statistical Manual of Mental Disorders (DSM) [244]. Consequently, it is imperative for forthcoming studies to delve into and elucidate the biomarkers and neuroimaging markers critical for PSD diagnosis, thereby enhancing diagnostic precision and mitigating the risks of misdiagnosis and suboptimal treatment.

In the evolving landscape of PSD management, the exploration of astrocytes as therapeutic targets heralds a novel paradigm in antidepressant strategies and broader interventions [67]. The role of cytokines as potential PSD predictors has gained prominence, acknowledging the variability in patients' inflammatory responses. Longitudinal and cross-sectional analyses of cytokine dynamics underscore the intricate link between oxidative stress and inflammation in PSD pathogenesis, enriching our understanding of its underlying mechanisms [257]. Additionally, conventional clinical inflammatory markers hold promise for tailoring PSD treatments to individual patient needs [245]. Given the integral role of oxidative stress in post-stroke inflammatory responses, a holistic treatment approach encompassing both antidepressant and antioxidant therapies is advisable. Evaluating depressive severity and oxidative stress metrics can guide the optimization of endogenous antioxidant treatment in ischemic stroke survivors, facilitating precise dosing decisions [246]. The choice of medication and treatment duration are pivotal considerations [247], with cognitive enhancers showing potential benefits in patients with mild to moderate depressive symptoms, albeit with limited efficacy in severe cases [248].

The pharmacological landscape for PSD is also being reshaped by insights into adiponectin's role in neuronal health, offering avenues to modulate inflammatory responses and alleviate depressive symptoms post-stroke through neuroplasticity regulation [249]. Furthermore, the gut-brain axis emerges as a critical factor in PSD, with gut microbiota imbalances linked to both psychiatric and gastrointestinal symptoms in PSD patients, positioning microbiota management as a potential therapeutic target [250]. rTMS represents an innovative rehabilitative approach with variable efficacy across PSD patients, underscoring the need for optimized stimulation protocols and targeted therapeutic strategies [251]. Additionally, mind-body exercises like Tai Chi and Qigong, bridging traditional and complementary medicine, have demonstrated efficacy in enhancing quality of life, motor function, daily living activities, and mitigating depressive symptoms in stroke survivors [122]. As the diagnostic criteria for PSD become more refined, strategies including astrocyte targeting, gut microbiota balance, rTMS and mind-body interventions are poised to offer new therapeutic directions. This broadened treatment spectrum promises a more integrated approach to PSD intervention, catering to the diverse needs of affected individuals.

### Conclusion

Depression ranks as a frequent sequela of stroke, rendering the elucidation of PSD pathogenesis imperative for its prevention, early detection, risk stratification, timely intervention, and tailored therapeutic approaches. Salient risk factors for PSD encompass gender, advanced age, pronounced disability, severe stroke episodes, antecedent depressive episodes, and cognitive deficits [252]. The multifaceted nature of PSD pathogenesis encompasses glial activation, HPA axis dysregulation, neurotransmitter and receptor alterations, diminished neuroplasticity and escalated oxidative stress, with the dimension of neuroinflammation warranting further exploration [192]. This discourse delineates neuroinflammation's pivotal role, interlinking it with diverse implicated mechanisms. Stroke-induced alterations in microglia and astrocytes emerge as critical in PSD evolution, modulating neurotransmitter dynamics, neuronal viability, and cytokine release, including pro-inflammatory IL-1β, IL-6, TNF-α, and anti-inflammatory IL-10 [253]. Stroke-triggered inflammation notably impacts HPA axis functionality [178] and curtails BDNF expression, thereby influencing synaptic plasticity [254]. Additionally, PSD's progression is influenced by mitochondrial anomalies, inflammation exacerbation [208, 255] and genetic variances [256], collectively shaping patients' cognitive and emotional landscapes.

Present-day PSD management predominantly employs antidepressants and psychotherapeutic modalities. An enriched comprehension of inflammatory pathways paves the way for potential anti-inflammatory and neurotrophic interventions. The prospect of utilizing non-steroidal anti-inflammatory drugs in PSD management is particularly promising. As molecular biology and neuroscientific domains progress, especially in genetics and epigenetics, insights into individual predispositions to PSD may unfold, heralding personalized treatment paradigms. Tailoring therapeutic regimens to a patient's genetic makeup and biomarkers represents a pivotal research and clinical trajectory. These advancements hold the promise of unveiling innovative treatment modalities, mitigating PSD prevalence and enhancing stroke survivors' quality of life.
